# Identification of the extracellular membrane protein ENPP3 as a major cGAMP hydrolase and innate immune checkpoint

**DOI:** 10.1016/j.celrep.2024.114209

**Published:** 2024-05-14

**Authors:** Rachel Mardjuki, Songnan Wang, Jacqueline Carozza, Bahar Zirak, Vishvak Subramanyam, Gita Abhiraman, Xuchao Lyu, Hani Goodarzi, Lingyin Li

**Affiliations:** 1 Department of Biochemistry, Stanford University, Stanford, CA 94305, USA; 2 ChEM-H Institute, Stanford University, Stanford, CA 94305, USA; 3 Arc Institute, Palo Alto, CA 94304, USA; 4 Department of Chemistry, Stanford University, Stanford, CA 94305, USA; 5 Department of Molecular and Cellular Physiology, Stanford University, Stanford, CA 94305, USA; 6 Department of Pathology, Stanford University, Stanford, CA 94305, USA; 7 Department of Urology, University of California, San Francisco, San Francisco, CA 94143, USA; 8 Helen Diller Family Comprehensive Cancer Center, University of California, San Francisco, San Francisco, CA 94158, USA; 9 Department of Biophysics & Biochemistry, University of California, San Francisco, San Francisco, CA 94143, USA; 10 Baker Computational Health Science Institute, University of California, San Francisco, San Francisco, CA 94143, USA; 11 Lead contact

## Abstract

2^’^3^’^-Cyclic guanosine monophosphate (GMP)-AMP (cGAMP) is a second messenger synthesized upon detection of cytosolic double-stranded DNA (dsDNA) and passed between cells to facilitate downstream immune signaling. Ectonucleotide pyrophosphatase phosphodiesterase I (ENPP1), an extracellular enzyme, was the only metazoan hydrolase known to regulate cGAMP levels to dampen anti-cancer immunity. Here, we uncover ENPP3 as the second and likely the only other metazoan cGAMP hydrolase under homeostatic conditions. ENPP3 has a tissue expression pattern distinct from ENPP1’s and accounts for all cGAMP hydrolysis activity in ENPP1-deficient mice. Importantly, we also show that, as with ENPP1, selectively abolishing ENPP3’s cGAMP hydrolysis activity results in diminished cancer growth and metastasis of certain tumor types in a stimulator of interferon genes (STING)-dependent manner. Both ENPP1 and ENPP3 are extracellular enzymes, suggesting the dominant role that extracellular cGAMP must play as a mediator of cell-cell innate immune communication. Our work demonstrates that ENPP1 and ENPP3 non-redundantly dampen extracellular cGAMP-STING signaling, pointing to ENPP3 as a target for cancer immunotherapy.

## In brief

Mardjuki et al. report ENPP3 as the only other major metazoan cGAMP hydrolase after ENPP1 under homeostatic conditions. Mice deprived of both innate immune checkpoints are protected from metastasis, and patients whose tumors have low levels of both have prolonged survival after anti-PD1 immunotherapy, pointing to a dual targeting strategy.

## Graphical Abstract

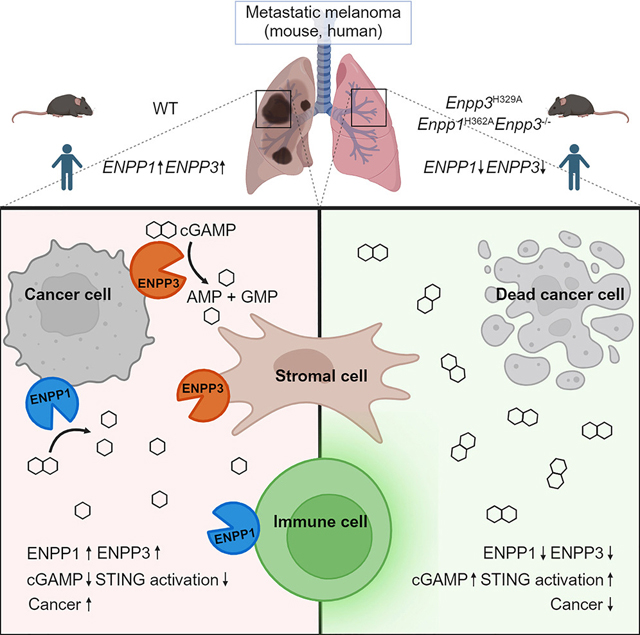

## INTRODUCTION

The presence of cytosolic double-stranded DNA (dsDNA) or DNA/RNA hybrids can be a sign of viral infection, cancer, aging, autoimmunity, or tissue damage. 2^’^3^’^-Cyclic guanosine monophosphate (cGAMP) is an innate immune second messenger synthesized by the enzyme cyclic GMP-AMP synthase (cGAS) upon detection of these molecular patterns.^1–5^ cGAMP activates its intracellular receptor, stimulator of interferon genes (STING), inducing type I interferons and other interferon-induced genes to mount an innate immune response to the detected threat.^2,6,7^ Besides signaling within the producing cell, cGAMP is also known to be exported from some producer cells, such as cancer cells, to activate the STING pathway in other nearby cell types.^8–12^ Activation of STING in responder cells, including fibroblasts, macrophages, and endothelial cells, elicits downstream anti-cancer^8,11,12^ and anti-viral immune responses but can also exacerbate systemic inflammation and autoimmunity.^13^ While STING activation in cancer cells can promote metastasis,^14,15^ STING activation in bystander cells of the tumor microenvironment delays primary tumor growth and metastasis and is responsible for the curative effect of radiation treatment.^8,12,16–21^ These nuances emphasize the importance of cell type- and tissue-specific regulation of cGAMP levels inside and outside the cell.

Ectonucleotide pyrophosphatase phosphodiesterase I (ENPP1) was reported 10 years ago as the first metazoan cGAMP hydrolase. ENPP1 is an extracellular type II transmembrane protein that regulates extracellular cGAMP levels in the context of cancer, infection, and local and systemic inflammation.^8,12,13,22–24^ Other second messengers, like cyclic guanosine monophosphate (cGMP) and cyclic adenosine monophosphate (cAMP), can be degraded by 11 different phosphodiesterase families (PDE1–PDE11), contributing to their cell-, tissue-, and context-specific regulation.^25^ However, it remains unclear whether there are other enzymes capable of degrading cGAMP that may act as a context-or tissue-specific regulator of STING activation.

Here, we set out to address this question by interrogating and isolating any remaining cGAMP hydrolase activity in mice lacking *Enpp1*. While some tissues in *Enpp1* knockout (*Enpp1*^−/−^) mice no longer have detectable cGAMP hydrolase activity, other tissues, like kidney, display a significant amount of remaining cGAMP hydrolase activity. We identified the responsible enzyme to be ENPP3, another extracellular enzyme and paralog of ENPP1. Mice harboring a point mutant of ENPP3 that specifically abolishes its cGAMP hydrolase activity are more resistant to both primary tumor growth and metastasis in certain cancers.

This study offers a target for cancer immunotherapy as well as mouse strains that can help elucidate extracellular cGAMP’s roles in health and disease. We detected no remaining activity in mice after disrupting the cGAMP hydrolase activity of both ENPP1 and ENPP3. We conclude that these two enzymes are likely the only two dominant mouse cGAMP hydrolases under homeostatic conditions, suggesting that all degradative regulation of cGAMP occurs extracellularly and that the predominant mode of signaling of cGAMP is through paracrine STING activation.

## RESULTS

### Identification and validation of ENPP3 as a cGAMP hydrolase

**Figure 1.**
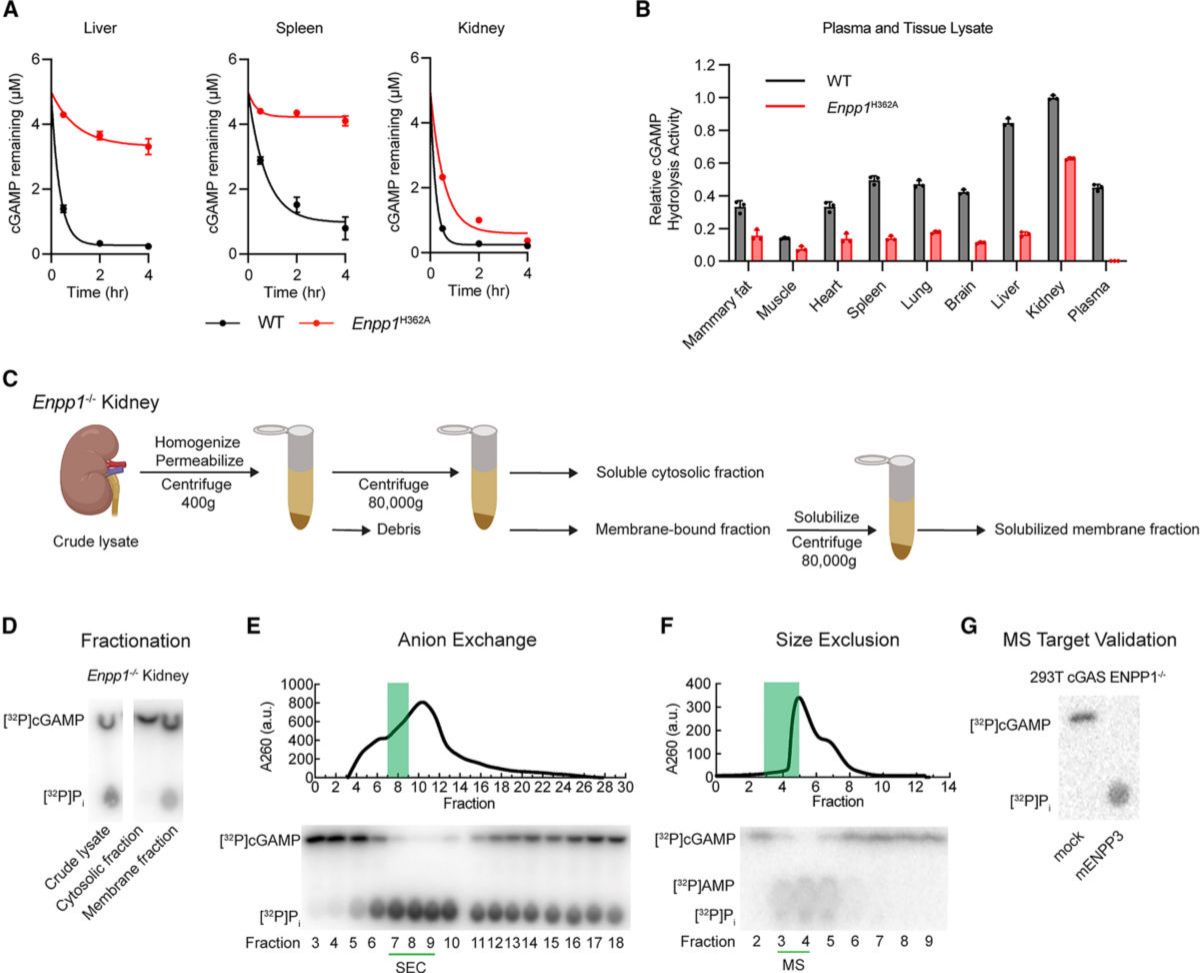
Identification and validation of ENPP3 as a cGAMP hydrolase (A) Example kinetic analysis of cGAMP degradation measured by thin-layer chromatography (*n* = 3, mean ± SD). (B) Rate constant of cGAMP degradation determined from example kinetic analysis. (C) Schematic for fractionation of ENPP3 from *Enpp1*^−/−^ mouse kidneys. (D) cGAMP degradation activity of fractions from (C). (E and F) Chromatography trace and cGAMP degradation activity from anion exchange and size exclusion chromatography. Green bars highlight fractions with the highest cGAMP hydrolysis activity/total protein concentration ratio. (G) cGAMP degradation by HEK293T cGAS *ENPP1*^−/−^ cells transfected with mENPP3.

*ENPP1* expression levels in patients with cancer have been shown to correlate with treatment response and prognosis,^12^ and cGAMP hydrolysis deficient *Enpp1*^H362A^ mice^13^ are routinely used to assess the contribution of cGAMP hydrolysis to regulating innate immune activity. However, it remains unclear whether enzymes besides ENPP1 can hydrolyze cGAMP. To answer this question, we harvested and homogenized various organs from wild-type (WT) and *Enpp1*^H362A^ mice to measure their cGAMP hydrolase activity (Figures 1A and 1B). As expected, tissues from which ENPP1 was initially purified and/or characterized in, including the liver, spleen, and plasma,^26^ had minimal cGAMP hydrolase activity remaining after *Enpp1* H362A mutation (Figures 1A and 1B). However, most *Enpp1*^H362A^ organs exhibited considerable residual cGAMP hydrolase activity, including the kidneys, mammary fat pads, lungs, and heart. Notably, the kidneys have both the most total activity and the highest overall cGAMP hydrolase activity-to-protein ratio (Figures 1A and 1B).

Therefore, we used *Enpp1*^–/–^ kidneys to isolate the unknown hydrolase(s) using serial fractionation (Figure 1C). We measured cGAMP hydrolase activity in the crude lysate, the cytosolic fraction, and the membrane fraction of *Enpp1*^–/–^ kidneys (Figures 1C and 1D). Only the membrane fraction, not the cytosolic fraction, had detectable levels of cGAMP hydrolase activity, suggesting the lack of intracellular cGAMP hydrolase activity in kidneys under the condition tested (Figure 1D). The final degradation product is inorganic phosphate (P_i_), evidenced by its lack of mobility on the thin-layer chromatography.

To further isolate the hydrolase(s), we subjected the solubilized membrane fraction to sequential anion exchange and size-exclusion chromatography (SEC) (Figures 1E and 1F). In the final size exclusion fractions, the degradation intermediate AMP became visible, suggesting that the more purified enzyme yields AMP and GMP as degradation products, which are hydrolyzed by any phosphatases in the lysate to P_i_. We analyzed the composition of size exclusion fraction 4 with the highest cGAMP hydrolase activity-to-protein ratio using mass spectrometry (MS). Size exclusion fraction 3, which has less cGAMP hydrolase activity than fraction 4, was also submitted for MS analysis.

Among the top hits, ENPP3, a paralog of ENPP1, was the fourth most abundant protein detected in fraction 4 (Table S1). ENPP3 was less abundant in fraction 3, further indication that ENPP3 is the unknown cGAMP hydrolase in *Enpp1*^–/–^ kidneys. Of the seven members of the ENPP family, ENPP1 is most closely related to ENPP3, both evolutionarily and structurally (Figures S1A and S1B).^27^ They are encoded only 60 kb apart on chromosome 6 and 10 of humans and mice, respectively. Like ENPP1, ENPP3 is a single-pass plasma transmembrane protein with an extracellular catalytic domain. To validate that ENPP3 is a cGAMP hydrolase, we transiently transfected mouse ENPP3 (mENPP3)-expressing construct into HEK293T cGAS *ENPP1*^–/–^ cells.^8^ The mENPP3-expressing cell lysate degraded cGAMP efficiently, whereas the untransfected control did not (Figure 1G). ENPP3 also exhibited a trace ability to degrade other bacterial cyclic dinucleotides, such as 3^’^3^’^-cGAMP and 3^’^3^’^-cyclic-di-AMP (3^’^3^’^-CDA) (Figure S1C), which is likely not physiologically significant. We investigated whether ENPP2, ENPP4, and ENPP5 could also cleave cGAMP, as the other members of the ENPP family known to cleave nucleotide-base substrates^28^ (Figure S1D). ENPP2 and ENPP5 did not exhibit any cGAMP hydrolase activity, while ENPP4 exhibited a trace amount of activity (Figures S1E–S1G). Together, these findings point to ENPP3 as the second major mammalian cGAMP hydrolase.

### ENPP3 is an efficient hydrolase of cGAMP at physiological pH

**Figure 2.**
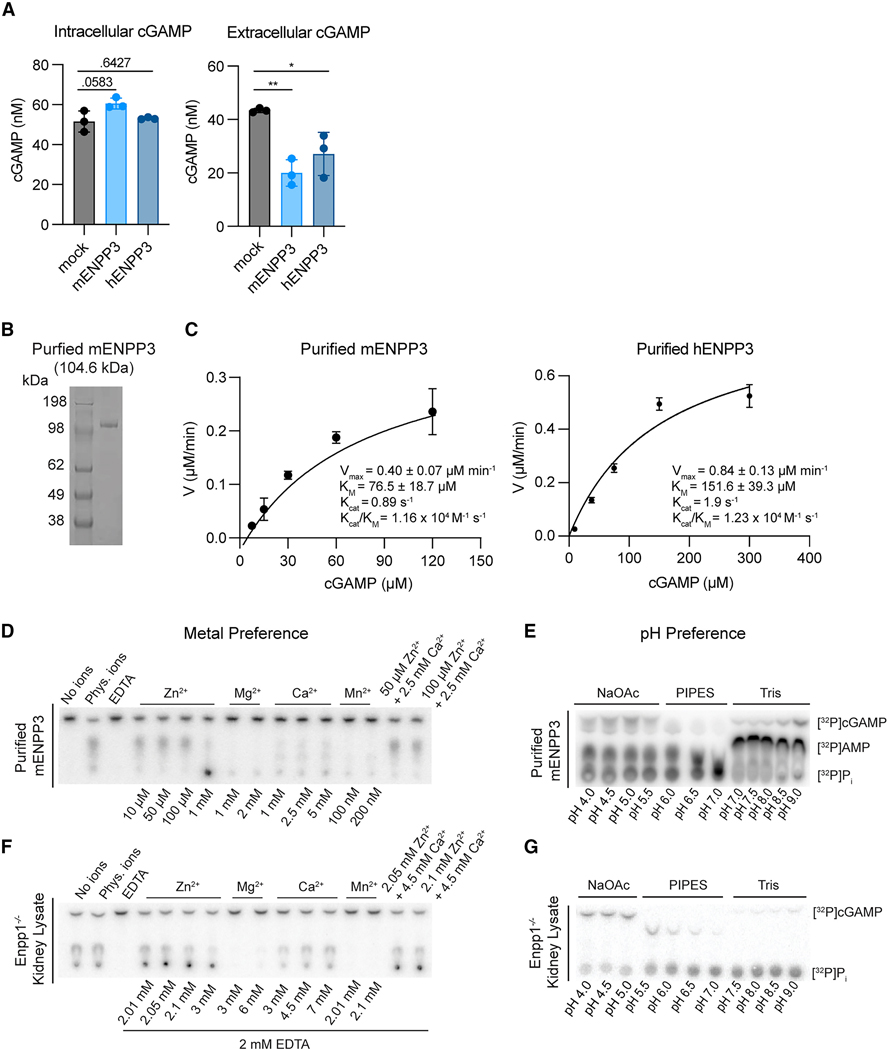
ENPP3 is an efficient hydrolase of cGAMP at physiological pH (A) cGAMP levels in HEK293T cGAS *ENPP1*^−/−^ cells transiently transfected with ENPP3. Significance was calculated by unpaired t test. (B) Coomassie gel of purified recombinant mENPP3. (C) Kinetics of cGAMP hydrolysis (*n* = 3). (D–G) Divalent ion (D and F) and pH preference (E and G) of *Enpp1*^−/−^ mouse kidney lysate and purified mENPP3. Significance was calculated as follows: ns, 0.1234; *p = 0.0332, **p = 0.0021, ***p = 0.0002. All data are mean ± SD.

Next, we sought to biochemically characterize ENPP3’s cGAMP hydrolase activity. ENPP1 has been reported to only degrade extracellular cGAMP.^8^ ENPP3, in contrast, has been shown previously to be active intracellularly in the lumen of the Golgi apparatus. In the Golgi, ENPP3 hydrolyzes uridine diphosphate N-acetylglucosamine (UDP-GlcNAc) to inhibit the function of the acetylglucosaminyltransferase GnT-IX and thereby affect global cell-surface protein glycosylation.^29^ Because cGAMP is synthesized in the cytosol and has not been shown to be transported into the Golgi, we hypothesize that only the extracellular plasma membrane-bound pool of ENPP3 degrades cGAMP. We transiently transfected mENPP3 and human ENPP3 (hENPP3) expressing constructs into HEK293T cGAS *ENPP1*^–/–^ cells. Like ENPP1, overexpression of mENPP3 and hENPP3 decreased extracellular cGAMP without affecting intracellular cGAMP, suggesting that only extracellular cGAMP is subject to ENPP3 degradation (Figure 2A).

Using purified recombinant mENPP3 (Figure 2B), we determined that ENPP3 efficiently hydrolyzes cGAMP (*K*_*M*_ = 76.5 ± 18.7 μM, *k*_cat_ = 0.89 s^–1^) at pH 9.0 (Figure 2C). hENPP3 also hydrolyzes cGAMP efficiently (*K*_*M*_ = 151.6 ± 39.3 μM, *k*_cat_ of 1.9 s^–1^, and *k*_cat_/*K*_*M*_ = 1.23 х 10^4^ M^–1^ s^–1^) (Figure 2C), albeit approximately 10-fold less efficient than hENPP1 (*K*_*M*_ = 15 μM, *k*_cat_ = 4 s^–1^, and *k*_cat_/*K*_*M*_ = 2.75 х 10^4^ M^–1^ s^–1^) under the same condition at pH 9.0.^26^

We then determined the optimal ion and pH conditions for degradation of cGAMP by ENPP3. It is known ENPP3 requires two Zn^2+^ ions for catalysis and Ca^2+^ ions for structural integrity.^30,31^ We confirmed this ion preference with recombinant mENPP3, where addition of Zn^2+^ and Ca^2+^, but not the other divalent ions (Mg^2+^ and Mn^2+^), increased mENPP3’s hydrolase activity (Figure 2D). ENPP1 peaks in activity at pH 9.0 ^26^; however, ENPP3 prefers a more neutral pH with a peak in activity around pH 7.0 (Figure 2E). As cGAMP and its degradation products migrate slightly differently on thin-layer chromatography under different buffer conditions, most notably in PIPES buffer, the peak in ENPP3 activity is most easily visualized by the disappearance of the cGAMP band at pH 7.0. We also found the same divalent cation and pH preference of 7.0 in the EDTA-treated *Enpp1*^–/–^ mouse kidney lysate, strongly suggesting that ENPP3 accounts for most, if not all, of the remaining cGAMP hydrolase activity in mice (Figures 2F and 2G).

### ENPP3 is the only other cGAMP hydrolase in mice

To determine ENPP3’s contribution to cGAMP hydrolase activity in tissues and to investigate ENPP3’s roles *in vivo*, we next sought to generate *Enpp3* knockout (*Enpp3*^–/–^) mice. However, like ENPP1,^32^ ENPP3 hydrolyzes extracellular ATP in addition to cGAMP. Additionally, ENPP3 hydrolyzes UDP-GlcNAc.^29^ While ENPP1 degrades ATP to maintain calcium homeostasis, ENPP1 degrades cGAMP to prevent systemic inflammation but inadvertently promotes cancer and viral infections.^12,13^ We created mice harboring an ENPP1 point mutation (*Enpp1*^H362A^), which abolishes ENPP1’s activity toward cGAMP but not ATP, allowing us to study the divergent roles of ENPP1 in regulating these two substrates.^12,13^ These findings demonstrate the need to isolate ENPP3’s roles in regulating cGAMP versus other substrates.

**Figure 3.**
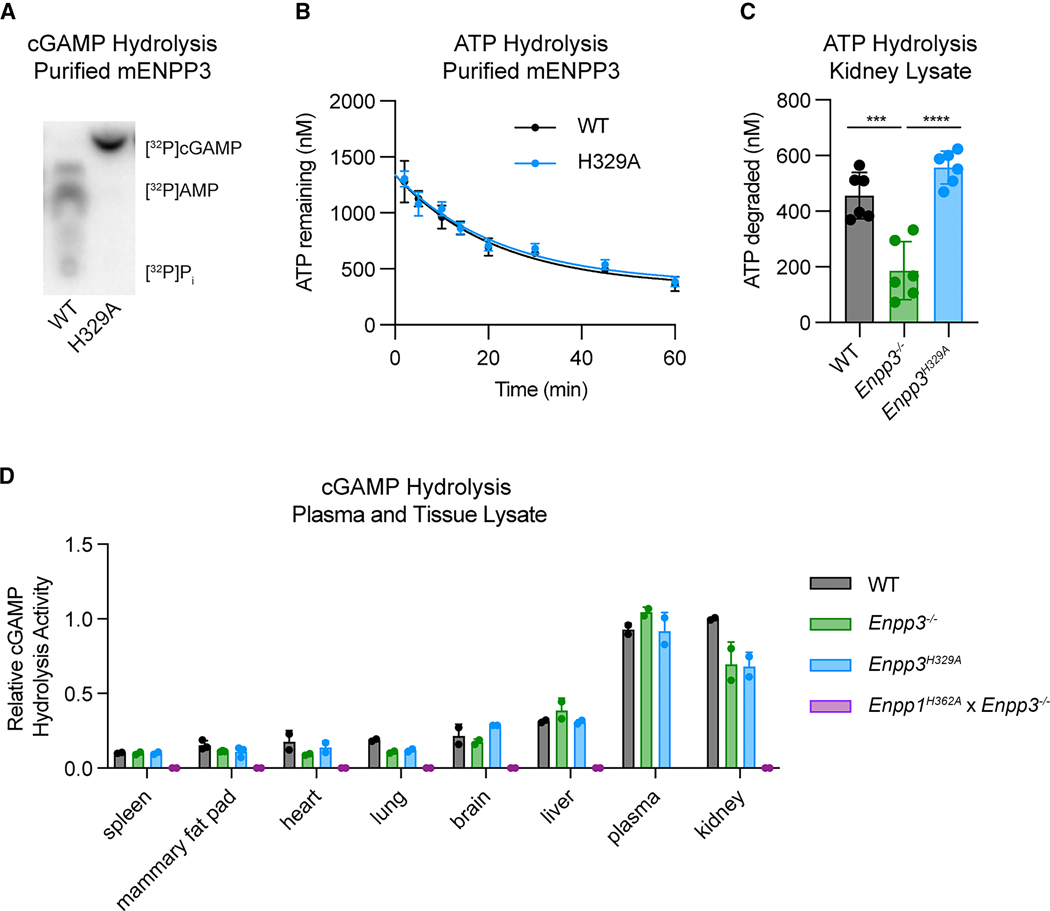
ENPP3 is the only other hydrolase in mice (A) cGAMP degradation by purified WT and H329A mENPP3. (B) Kinetics of ATP degradation by purified WT and H329A mENPP3 (*n* = 3). (C) ATP degradation by kidney lysates from mice of the indicated genotypes (*n* = 6). Significance was calculated by unpaired t test. (D) Rate constant of cGAMP degradation in organ lysate determined as in Figure 1A *n* = 2 per genotype except mammary fat pad (*n* = 3). Significance was calculated as follows: ns, 0.1234; *p = 0.0332, **p = 0.0021; ***p = 0.0002. All data are mean ± SD.

As ENPP1 and ENPP3 have similar catalytic sites, we identified H329A ENPP3 as the analogous mutation to H362A ENPP1. *In vitro* studies confirmed that the H329A mutation in mENPP3 completely abolishes its hydrolase activity toward cGAMP while preserving its activity toward ATP (Figures 3A and 3B). The H329A mENPP3 mutation also retains its hydrolase activity toward UDP-GlcNAc (Figure S2).

Using CRISPR-based homologous recombination, we generated *Enpp3*^–/–^ and homozygous *Enpp3*^H329A^ mice on the C57BL/6 genetic background (Figures S3A–S3E). Kidney lysate from *Enpp3*^H329A^ and WT mice degraded ATP similarly, indicating that ENPP3^H329A^ is still capable of degrading ATP. However, *Enpp3*^–/–^ kidney lysate degraded less than half the ATP of WT mice, indicating that ENPP3 ATP hydrolysis activity was abolished as expected in *Enpp3*^–/–^ mice (Figure 3C). In tissues with considerable cGAMP hydrolase activity remaining after *Enpp1*^–/–^, such as kidney, lung, heart, and mammary fat pad (Figure 1B), we observed decreased cGAMP hydrolase activity from both *Enpp3*^–/–^ and *Enpp3*^H329A^ mice compared with the WT (Figure 3D). In contrast, in the plasma, liver, brain, and spleen, where cGAMP hydrolase activity was primarily attributable to ENPP1 (Figure 1B), *Enpp3*^–/–^ and *Enpp3*^H329A^ did not impact cGAMP hydrolase activity (Figure 3D). We found that using H329A, the analogous separation-of-function mutation to ENPP1’s H362A, we were able to selectively abolish ENPP3 cGAMP hydrolase activity *in vitro* and *in vivo*. To better visualize the contributions of ENPP1 and ENPP3 in various organs, we normalized the data from Figures 1B and 3D and displayed them in a stacked bar graph (Figures S3F and S3G).

To definitively determine whether ENPP1 and ENPP3 are the only cGAMP hydrolases in mice, we generated mice lacking both ENPP1 and ENPP3 cGAMP hydrolysis activity. *Enpp1* and *Enpp3* are linked genes due to their proximity, so we could not cross our *Enpp1*^H362A^ mice to our *Enpp3*^H329A^ mice to generate *Enpp1*^H362A^ х *Enpp3*^H329A^ mice. We thus performed a second round of CRISPR-based editing of *Enpp3* on the *Enpp1*^H362A^ background. The rate of successful homology-directed repair in mouse embryos has been reported to be as low as 1%–2%,^33^ so, with only 10 founder mice, we were unable to obtain any *Enpp1*^H362A^ х *Enpp3*^H329A^ mice and instead generated homozygous *Enpp1*^H362A^ х *Enpp3*^−/−^ mice. Tissues harvested from *Enpp1*^H362A^ х *Enpp3*^−/−^ mice displayed no detectable residual cGAMP hydrolase activity, confirming that ENPP3 is the only other dominant hydrolase in mice aside from its paralog ENPP1 (Figures 3D and S4).

### ENPP1 and ENPP3 have different expression patterns that influence their effect on cancer

**Figure 4.**
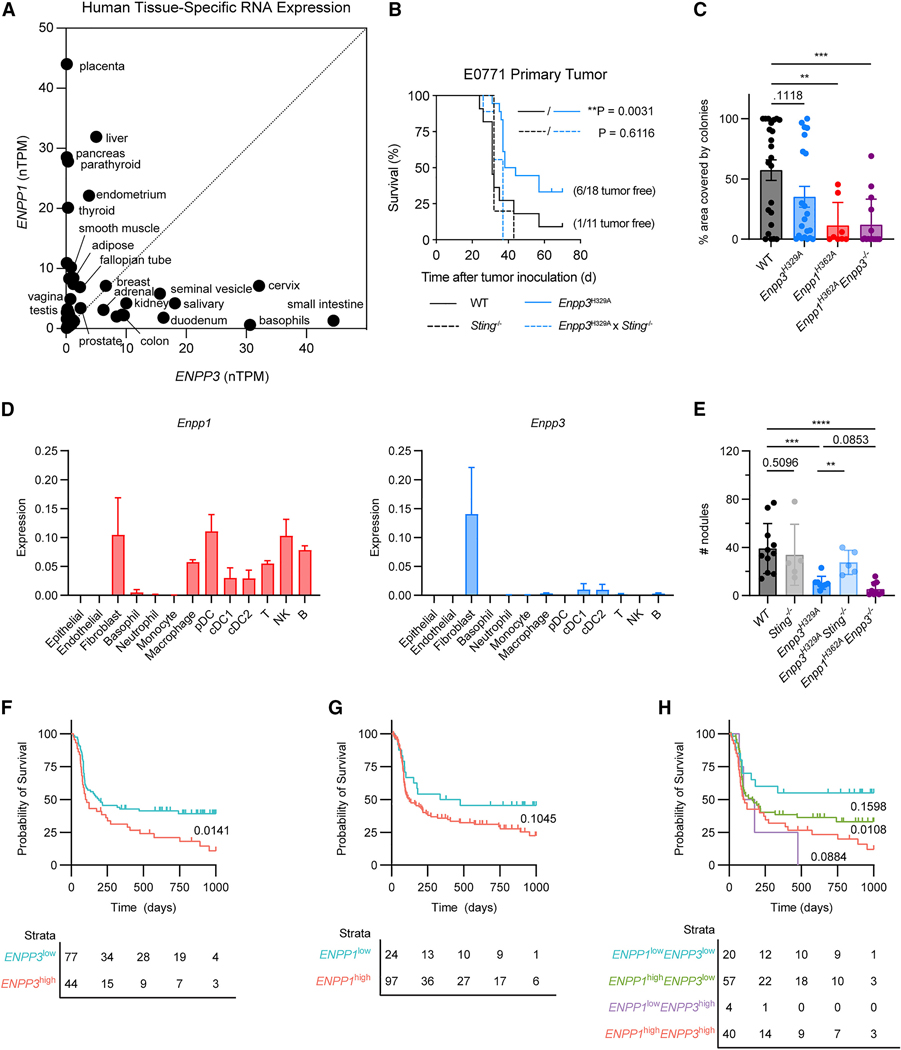
ENPP3 promotes tumor growth and metastasis by dampening the innate immune cGAMP-STING signaling pathway (A) Expression of ENPP1 and ENPP3 in human tissue. Data are from the Human Protein Atlas (https://www.proteinatlas.org/). (B) Survival curves for orthotopically injected E0771 breast cancer cells in genotypes: WT (*n* = 11), *Sting*^−/−^ (*n* = 5), *Enpp3*^H329A^ (*n* = 18), and *Enpp3*^H329A^*Sting*^−/−^ (*n* = 9). (C) Metastasis of intravenously injected E0771.LMB breast cancer cells in genotypes: WT (*n* = 23), *Enpp3*^H329A^ (*n* = 21), *Enpp1*^H362A^ (*n* = 8), *Enpp1*^H362A^*Enpp3*^−/−^ (*n* = 15). (D) Bar graphs of expression of *Enpp1* and *Enpp3* in the indicated cell types from single-cell RNA sequencing of 4T1 mouse breast cancer pulmonary metastasis. (E) Metastasis of intravenously injected B16-F10 melanoma cells in genotypes: WT (*n* = 11), *Sting*^−/−^ (*n* = 5), *Enpp3*^H329A^ (*n* = 8), *Enpp3*^H329A^*Sting*^−/−^ (*n* = 5), *Enpp1*^H362A^*Enpp3*^−/−^ (*n* = 9). (F–H) Comparison of progression-free survival rate of patients with ENPP1 or ENPP3 expression. The numbers of remaining patients are listed under the graphs. Significance in graphs was calculated by log rank Mantel-Cox test. Significance was calculated as follows: ns, 0.1234; *p = 0.0332, **p = 0.0021, ***p = 0.0002. All data are mean ± SD.

While ENPP1 and ENPP3 have similar biochemical functions, they have distinct tissue expression patterns. From Human Protein Atlas data, we found that, while *ENPP1* and *ENPP3* are expressed to similar levels in tissues such as breast and prostate, their expression levels are orthogonal in many other tissues. For example, the placenta and liver predominantly express *ENPP1* while the small intestine and basophils predominantly express *ENPP3* (Figure 4A).^27^ Furthermore, ENPP1 accounts for virtually all of the cGAMP hydrolase activity in mouse plasma; *Enpp1*^−/−^ mouse plasma no longer has any detectable cGAMP hydrolase activity (Figures 1B and 3D).

In disease settings, *ENPP1* is known to be highly expressed in many human cancer types, including breast cancer and glioblastoma.^34,35^ We and others have shown previously that ENPP1 degrades cancer-produced extracellular cGAMP to dampen downstream STING signaling in stromal cells and tumor-infiltrating immune cells.^8,12,23^ Here, we asked whether *ENPP3* is also highly expressed in some cancer types. We analyzed patient data from The Cancer Genome Atlas (TCGA) to compare *ENPP1* and *ENPP3* expression across cancer types (Figure S5A). Similar to the distinct expression patterns of *ENPP1* and *ENPP3* in normal human tissues, they are also differentially expressed in human cancers. In some cancer types, such as advanced thyroid carcinoma (THCA), only *ENPP1* is expressed; in others, such as colon adenocarcinoma (COAD), kidney renal clear cell carcinoma (KIRC), prostate adenocarcinoma (PRAD), and uterine corpus endometrial carcinoma (UCEC), *ENPP3* is uniquely expressed, and in breast invasive carcinoma (BRCA), though most tumors largely express *ENPP1*, a subset expresses both *ENPP1* and *ENPP3*. This differential expression pattern is also evident in the protein staining of patient tumor samples for *ENPP1* and *ENPP3* as well, where, in liver adenocarcinoma and endometroid carcinoma, one enzyme is highly present but the other absent (Figures S5B and S5C). Finally, expression of *ENPP1* and *ENPP3* can differ by patient (Figures S5D and S5E) and by spatial localization (Figure S5F) within the same cancer types as well.

Given that ENPP1 and ENPP3 are co-expressed in some human BRCA tumors, we hypothesized that ENPP3, like ENPP1, is an innate immune checkpoint that promotes breast cancer by dampening the cGAMP-STING pathway in orthotopic murine breast cancer models.^12^ Indeed, in the orthotopic E0771 breast cancer model, mice harboring the cGAMP hydrolysis-deficient *Enpp3* gene (*Enpp3*^H329A^) were significantly more resistant to tumor initiation and growth than WT mice: 6 of 18 mice remained tumor free by the end of the 10-week study, compared to 1 of 11 tumor-free WT mice (Figure 4B). This effect was entirely STING dependent, as *Enpp3*^H329A^ × *Sting*^−/−^ mice have tumor progression similar to WT and *Sting*^−/−^ mice. These results mirror our previous observation that mice harboring the cGAMP hydrolysis-deficient *Enpp1* gene (*Enpp1*^H362A^) exhibit STING-dependent delayed primary tumor growth in the same orthotopic E0771 breast cancer model.^12^

We have demonstrated previously that the expression of *Enpp1* is deterministic of breast cancer metastasis into the lungs and other organs.^12^ We thus hypothesized that ENPP3 would not affect breast cancer lung metastasis despite its similar activity to ENPP1 in mouse lungs (Figures S3F and S3G). In this experiment, E0771.LMB puro-resistant (E0771.LMB.PuroR) breast cancer cells are introduced into circulation through tail vein injections to induce pulmonary metastasis. As expected, unlike what we observed with *Enpp1*^H362A^ mice, we did not observe appreciable differences in lung metastatic burden between *Enpp3*^H329A^ and WT mice 30 days after cancer cell injection (Figure 4C). As ENPP3 does not affect breast cancer lung metastasis, we hypothesized that metastasis in the *Enpp1*^H362A^ х *Enpp3*^−/−^ mice would be similar as that of *Enpp1*^H362A^ mice. Indeed, *Enpp1*^H362A^ and *Enpp1*^H362A^ х *Enpp3*^−/−^ mice reduced metastatic burden similarly.

To understand the immunological mechanism of the differential effect of ENPP1 and ENPP3 in breast cancer lung metastasis, we mined our previous single-cell RNA sequencing dataset of 4T1 mouse breast cancer pulmonary metastasis to see how *Enpp1* and *Enpp3* are differentially expressed in the tumor microenvironment. In this dataset, *Enpp1* is highly expressed on infiltrating immune cells such as macrophages, plasmacytoid dendritic cells (pDCs) and natural killer (NK) cells as well as tumor-associated fibroblasts (Figure 4D).^12^ However, *Enpp3* is not expressed on these infiltrating immune cells but, rather, on fibroblasts. Breast cancers are known to be heavily infiltrated by immune cells, perhaps explaining the major role of ENPP1 in breast cancer metastasis.^36^

Given fibroblasts’ general role in supporting tumor growth,^37^ we hypothesize that ENPP3 might play a bigger role in cancer types with less infiltration of immune cells. To test this hypothesis, we chose the B16-F10 melanoma model, which is known to have fibroblast involvement but very little immune infiltration.^38^ B16-F10 melanoma cells were introduced into circulation through tail vein injections to induce pulmonary metastasis. Thirty days after inoculation, we found that *Enpp3*^H329A^ mice had significantly fewer surface lung nodules than WT mice (Figure 4E). The effect is STING dependent, as *Enpp3*^H329A^ х *Sting*^−/−^ mice have similar numbers of lung nodules as WT and *Sting*^−/−^ mice. These data suggest that, in certain cancer types, ENPP3, like ENPP1, is an innate immune checkpoint that promotes metastasis by degrading cGAMP and dampening STING pathway activation. Given that we still observed metastasis in *Enpp3*^H329A^ mice, we hypothesized that *Enpp1*^H362A^ х *Enpp3*^−/−^ mice lacking both ENPP1 and ENPP3 activity might be more protected than the ENPP3 mutant mice alone. Indeed, *Enpp1*^H362A^ х *Enpp3*^−/−^ mice have fewer metastases than *Enpp3*^H329A^ mice (Figure 4E).

Given ENPP3’s pro-metastasis role in the mouse model, we analyzed previously published data from a cohort of patients with melanoma (*n* = 144) treated with anti-PD1 immune checkpoint blockade.^39^ Strikingly, patients with tumors with higher levels of ENPP1 or ENPP3 expression had lower overall progression-free survival rates than patients with tumors with lower levels of expression (Figures 4F and 4G). In addition, the effects of ENPP1 and ENPP3 are additive; patients with tumors expressing lower levels of both ENPP1 and ENPP3 had a better chance of progression-free survival than patients with tumors expressing higher levels of both enzymes (Figure 4H).

## DISCUSSION

cGAMP is the most recently discovered mammalian second messenger, and despite growing evidence of its cornerstone role in cancer immunology, anti-viral responses, and autoimmunity, our understanding of its physiology in complex tissues and systems remains incomplete. Other second messengers, like cAMP and cGMP, have 11 phosphodiesterases (PDEs), making ENPP1 unlikely to be the sole metazoan hydrolase of cGAMP. Here we report the discovery of ENPP3 as a cGAMP hydrolase. Our *Enpp1*^H362A^ х *Enpp3*^−/−^ mice have no detectable cGAMP hydrolase activity, indicating that ENPP1 and ENPP3 are the dominant hydrolases of cGAMP.

We cannot rule out the possibility that other cGAMP hydrolases could be induced or activated upon detection of a pathogen or other stimulus. Indeed, while this paper was under review, the discovery of sphingomyelin phosphodiesterase acid-like 3A (SMPDL3A), a third cGAMP hydrolase, was reported.^40^ SMPDL3A is a secreted enzyme whose expression is induced by liver × receptor ligands. This induction is required for the activity of SMPDL3A, as we could not detect evidence of its activity despite thorough investigation of our *Enpp1*^H362A^ х *Enpp3*^−/−^ mice (Figures 3D and S4). Therefore, the physiological relevance of degradation of cGAMP by SMPDL3A, particularly in the cancer setting, remains unknown.

Other second messengers, such as cAMP and cGMP, are reported to have only intracellular function and regulation. In contrast, cGAMP has only been found to have extracellular regulation with many known cGAMP transporters^9,11,21,41–44^ and hydrolases that are all extracellular. It is thus reasonable that the majority of cGAMP regulation would occur in the extracellular space. We have shown that the presence of cytosolic DNA leads to the production of extracellular cGAMP to mediate immunity in a STING-dependent manner.^8,11,13^ We propose a model in which cGAMP’s main physiological function is that of a paracrine immunotransmitter,^8,12,13,45^ and the differential expression of *Enpp1* and *Enpp3* regulates how much cGAMP cells emit and receive.

Our studies of primary breast tumor growth and melanoma lung metastasis suggest that, like ENPP1, ENPP3 is an innate immune checkpoint that should be targeted to boost anti-cancer immunity. ENPP1 and ENPP3 are similar but distinct enzymes regarding their expression patterns and biochemical properties. Similar distinct expression patterns have been exploited in PDE inhibition, where drugs targeting different PDEs have different indications depending on their tissue expression. PDE4 is predominantly expressed in the lungs, and its inhibitor, roflumilast, treats severe chronic obstructive pulmonary disease (COPD); PDE5 is predominantly expressed in vascular and trabecular smooth muscles, and PDE5 inhibitors treat erectile dysfunction.^46,47^ In addition, ENPP3 has activity in a broad pH range compared to ENPP1, which may provide ENPP3 with an outsized advantage in the acidic tumor microenvironment of some tumor types. Inhibitors of ENPP1 are currently in clinical trials (ClinicalTrials.gov: NCT05978492 and NCT06063681) as a potential treatment for cancer. The reduced breast cancer and in *Enpp1*^H362A^ × *Enpp3*^−/−^ mice (Figures 4C and 4E) indicate that dual inhibitors targeting both ENPP1 and ENPP3 should be developed as potential immunotherapy that may benefit a larger population of patients with cancer.

### Limitations of the study

Here we generated mice with *Enpp1* and *Enpp3* mutations that render the enzymes specifically inactive for cGAMP hydrolysis. As the two genes are linked by proximity, they cannot be crossed, and we were only able to generate *Enpp1*^H362A^ х *Enpp3*^−/−^ mice. The *Enpp1*^H362A^ х *Enpp3*^−/−^ mice showed greater resistance to melanoma metastasis, but as *Enpp3* was completely ablated, we cannot exclude the role of extracellular ATP in increasing resistance to metastasis. Future work using *Enpp1*^H362A^ х *Enpp3*^−/−^х *Sting*^−/−^ can formally test the potential contribution of ATP in metastasis resistance.

## STAR★METHODS

### RESOURCE AVAILABILITY

#### Lead contact

Further information and requests for resources and reagents should be directed to and will be fulfilled by the corresponding author, Dr. Lingyin Li (lingyin@arcinstitute.org).

#### Materials availability

All materials generated in this study are available from the lead contact upon request.

#### Data and code availability

This study does not report original code or data.

### METHOD DETAILS

#### Fractionation of *Enpp1*^−/−^ mouse kidney

Kidney from *Enpp1*^−/−^ mouse was isolated and flash-frozen in liquid nitrogen. On day of homogenization, kidney was thawed in 1 mL of hypotonic buffer (10 mM Tris pH 7.5, 150 mM sucrose including EDTA-free cOmplete protease inhibitor (Roche)) and homogenized using bead mill (5 cycles, 40 s on, 40 s off, Fisherbrand). Following homogenization, NaCl was supplemented to 150 mM and Tris to 50 mM pH 7.5. Digitonin was added to a final concentration of 0.25% to permeabilize the plasma membrane but leave the organelles intact. Following a 30–60 min incubation with rotation at 4C, all soluble proteins were thus separated by centrifugation at 80,000g for 1 h at 4C. The remaining pellet was solubilized using 1.5–2% NP-40 in 50 mM Tris pH 7.5 and 150 mM NaCl for 2–3 h with rotation at 4C. A second centrifugation at 80,000g for 1 h at 4C was performed to isolate the solubilized membrane proteins.

The supernatants from the centrifuge spins were diluted 50x into buffer containing 20 mM Tris pH 7.5 and 0.1% NP-40 prior to anion exchange on a 1-mL HiTrap Q column (Cytiva). The elution was performed from 0 mM to 500 mM NaCl in 20 mM Tris pH 7.5 with 0.1% NP-40 over a gradient involving 30 column volumes.

Following anion exchange, each fraction was evaluated for cGAMP degradation activity. 8mL of each fraction from AEX was supplemented with 1 μL of 10x physiological ion buffer, radioactive cGAMP and cold cGAMP to 5 μM final concentration for a total volume of 10 μL. Cold cGAMP was purified as previously described. Radiolabeled cGAMP was purified as described in the section immediately following. At 1× concentration, physiological ion buffer contained 15 μM ZnCl_2_, 2.5 mM CaCl_2_, 1mM MgCl_2_, 100 nM MnCl_2_, 5 mM KCl, 150 mM NaCl, and 50 mM Tris pH 7.5. The reactions were incubated at room temperature overnight, after which the radioactive TLC described above was performed to evaluate the extent of cGAMP degradation in each fraction.

The fractions with the highest activity from AEX identified via TLC were pooled, concentrated and subjected to size exclusion on a Superose 6 Increase 10/300 GL column (Cytiva). Each fraction from size exclusion was again evaluated for activity. The entire fraction was subject to MS analysis at the Vincent Coates Foundation Mass Spectrometry laboratory, Stanford University Mass Spectrometry. 1555 proteins were identified in total (Supplementary DataSet). ENPP3 ranked #4 on the list.

#### Synthesis and purification of ^32^P-GAMP

Radiolabeled ^32^P-ATP (3000 Ci/mmol) used for synthesis of ^32^P-cGAMP was purchased from PerkinElmer. 1 μM purified sscGAS was incubated with 20 mM Tris-HCl pH 7.4, 2 mM ATP, 2 mM GTP, 20 mM MgCl2, and 100 μg/mL herring testis DNA (Sigma) for 1–3 days. Resulting ^32^P-cGAMP was purified via preparatory TLC as previously described.^1^

#### ^32^P-cGAMP degradation TLC assay

To test for the presence of cGAMP hydrolases, 1 nM ^32^P-cGAMP and 5 μM cGAMP were incubated in fractions from anion exchange and size exclusion for 1–20 h supplemented with physiological levels of ions (15 μM ZnCl_2_, 2.5 mM CaCl_2_, 1 mM MgCl_2_, 5 mM KCl, 100 nM MnCl_2_, 150 mM NaCl) with 50 mM Tris pH 7.5. Plasma was collected from whole blood collected in heparin-coated BD Microtainer, centrifuged at 10,000g for 10 min at 4C. Plasma was diluted to 60% in reactions to reduce streaking of cGAMP on TLC.

1–2 μL from the reaction was spotted on a silica TLC plate (Millipore Sigma) and allowed to dry for a minimum of 15 min. The TLC was developed in a solvent containing 85% ethanol and 5 mM NH_4_HCO_3._ Plates were dried and exposed to a Storage Phosphor Screen (Cytiva) overnight before being imaged with an Amersham Typhoon 5 Imager (Cytiva).

#### ATP degradation assay

ATP assays were conducted in 15 μL total volumes in a 384 well plate. 5 μM ATP were combined with organ lysate (0.1%) or recombinant ENPP3 (5 nM) and assay buffer (50 mM Tris pH 7.5, 150 mM NaCl, 500 μM CaCl_2_, 10 μM ZnCl_2_). Reactions were started at indicated times and ended simultaneously by heating at 95C for 10 min. 10 μL of each reaction were transferred to a white-walled 384 well plate, mixed with CellTiterGlo (5 μL), and luminescence was read after 15 min on a Tecan Spark plate reader.

#### Degradation of cyclic dinucleotides (CDNs)

To test for degradation of 3^’^3^’^-cGAMP and 3^’^3^’^-cDA by ENPP3, 5 μM of each respective CDN was incubated with 25 nM of mENPP3 for the time indicated. Reactions were run in 15 μL volumes with 50 mM Tris pH 7.5, 150 mM NaCl, 500 μM CaCl_2_, 10 μM ZnCl_2_. Resultant AMP from each reaction was quantified by our previously published assay cGAMP-Luc assay.^10^ In brief, AMP is converted enzymatically by polyphosphate: AMP phosphotransferase and myokinase to ATP. The resultant ATP can be enzymatically converted to light using the CellTiterGlo (Promega) reagent and quantified on plate reader (Tecan Spark).

#### Cloning of full-length mENPP3

The DNA sequence encoding mouse ENPP3 (mENPP3) was cloned from a WT C57BL/6J mouse kidney. RNA from the mouse kidney was a generous gift from Wei Wei in the laboratory of Prof. Jon Long. A cDNA library was generated from the RNA using the iScript cDNA synthesis kit (BioRad). Full-length ENPP3 was PCR amplified using Phusion High-Fidelity DNA polymerase (Thermo). The PCR product was inserted into a pcDNA3 vector using Gibson assembly containing a TEV site followed by a TARGET-tag,^2–4^ 12 histidine residues and a stop codon as previously published.^4,5^

Primers for amplification of full length mENPP3 from cDNA:

Fwd: CTACGGGAACAATGGATTCCAG.

Rev: CATTCAAATAATGGTTTCAAACGTGGGCAGATACGTC.

Primers for amplification of full length mENPP3 for Gibson assembly:

Fwd: GGAGACCCAAGCTGGCTAGTTAAGCTTGCCATGGATTCCAGGCTAGCATTAGCCACAGAG.

Rev: CTTACCTTGGAAGTACAGGTTCTCTCTAGAAATAATGGTTTCAAACGTGGGCAG.

#### Cloning of secreted mENPP3

cDNA from above was used to amplify all of mENPP3 except for its cytosolic and transmembrane domains (nucleotide 136–2622). The mENPP2 signal sequence was attached to the 5^’^ end of the mENPP3 sequence using successive rounds of PCR. The secreted mENPP3 construct was then inserted into a pcDNA3 vector using Gibson assembly containing a C-terminal TEV site followed by a TARGET-tag,^2–4^ 12 histidine residues and a stop codon as previously published.^4,5^

Primers for addition of mENPP2 signal sequence:

Fwd Primer 1: CTCTGCTTAGGAAGGAAACCTGAAGAACAAGGCAG.

Fwd Primer 2: GGTAATATCCTTGTTCACTTTTGCCATCGGCGTCAATCTCTGCTTAGGAAGGAAACC.

Fwd primer 3: GCCATGGCAAGACAAGGCTGTTTCGGGTCATACCAGGTAATATCCTTGTTCAC.

Rev: AATAATGGTTTCAAACGTGGGCAGATACGTC.

Primer for amplification of secreted mENPP3 for Gibson assembly:

Fwd: TATAGGGAGACCCAAGCTGGCTAGTTAAGCTTGCCATGGCAAGACAAGGCT.

Rev: CTTACCTTGGAAGTACAGGTTCTCTCTAGAAATAATGGTTTCAAACGTGGGCAGA.

#### Cloning of mENPP3 mutations

The H329A variant was introduced via site-directed mutagenesis. Mutated plasmids were cloned using pfuTurbo DNA polymerase (Agilent) and parent plasmids were degraded by DpnI (NEB). All plasmids were transformed into DH5α cells.

Primers for generation of H329A mutation.

Fwd: CCTGATTCTGCAGGGGCGTCGAGTGGACCAGTC.

Rev: GACTGGTCCACTCGACGCCCCTGCAGAATCAGG.

#### Cell culture

The HEK 293T cell line was procured from ATCC. The HEK 293T cGAS *ENPP1*^−/−^ cell line originates from previously published work.^4^ This cell line, which overexpresses cGAS on an ENPP1^−/−^ background, was previously generated for the purpose of studying cGAMP export in 293T cells. Here, the cell line was chosen for its lack of cGAMP degradation following ablation of *ENPP1*. The B16-F10, EO771 and EO771.lmb cell lines for cancer experiments were procured from ATCC.

All cell lines were maintained in a 5% CO2 incubator at 37°C. 293T and B16-F10 cell lines were maintained in DMEM (Corning Cell-gro) and the EO771 cell lines were maintained in RPMI (Corning Cellgro) supplemented with 10 mM HEPES (Gibco). All cell lines were supplemented with 10% FBS (Atlanta Biologics, v/v) and 100 U/mL penicillin-streptomycin (ThermoFisher).

#### Quantification of cGAMP

293T cGAS ENPP1^−/−^ cells were plated in tissue culture treated plates coated with 2% PurCol (Advanced BioMatrix). 24 h after transient transfection with mouse or human ENPP3 via Fugene 6 (Promega), the media was gently removed and replaced with serum-free DMEM supplemented with 1% insulin-transferrin-selenium-sodium pyruvate (ThermoFisher) and 100 U/mL penicillin-streptomycin. 24 h after changing to serum-free media, media and cells were collected and centrifuged at 1000 rcf for 5 min at room temperature. Cells were washed with PBS. Cells were lysed in M-PER Mammalian Protein Extraction Reagent (Thermo Scientific) and analyzed for cGAMP content alongside media using the 2^’^3^’^ cGAMP ELISA kit (Cayman Chemical).

#### Quantification of degradation of UDP-GlcNAc

0.8 and 4 nM of recombinant mENPP3 was incubated with 10 μM UDP-GlcNAc in buffer containing 50 mM Tris pH 7.5, 150 mM NaCl, 0.5 mM CaCl_2_, 10 μM ZnCl_2_, 0.1% NP-40 at room temperature in 20 μL reactions. The reaction was inactivated at 95 °C at the indicated timepoints. Following the reaction, the UMP/CMP-Glo Glycosyltransferase Assay (Promega) was used to quantify the resulting UMP. Data was normalized to enzyme concentration used.

#### Characterization kinetics of ENPP3

To characterize the biochemical kinetics of ENPP3, 7.5 nM of either mouse or human ENPP3 was incubated with the indicated concentrations of cGAMP spiked with ^32^P-cGAMP. Mouse ENPP3 was purified as described in the section immediately following; human ENPP3 was purchased from AcroBiosystems (#EN3-H52H4). Reactions were performed in buffer containing 20 mM Tris pH 9, 50 mM NaCl, 1 mM CaCl_2_, and 100 μM ZnCl_2_. Timepoints of 0, 10, 20, 30, 45, 60, 75, 90, 120 and 160 min were taken. At each timepoint, 1.5 μL from the reaction was spotted on a silica TLC plate (Millipore Sigma) and allowed to dry for a minimum of 15 min. The TLC was developed in a solvent containing 85% ethanol and 5 mM NH_4_HCO_3._ Plates were dried and exposed to a Storage Phosphor Screen (Cytiva) overnight before being imaged with an Amersham Typhoon 5 Imager (Cytiva).

#### Purification of secreted ENPP3 and ENPP1

Supernatant of ENPP1 or ENPP3-transfected 293T cells were supplemented with His-Pur cobalt resin (ThermoFisher), imidazole to 10 mM, NaCl to 150 mM, Tris to pH 7.5 and cOmplete protease inhibitor cocktail (Roche). Following incubation for 1 h at 4°C, resin was washed with 15 column volumes of buffer made of 50 mM Tris pH 7.5, 150 mM NaCl (1× TBS) and 10 mM imidazole. A second wash of 15 column volumes of buffer made of 20 mM imidazole in 1× TBS was performed. ENPP3 or ENPP1 was eluted with 10 column volumes of 300 mM imidazole in 1× TBS. Buffer exchange to remove imidazole from the mixture was performed in centrifugal filter with a 10-kDa molecular mass cutoff (Millipore). Following quantification via micro-BCA assay (Thermo Fisher), ENPP3 or ENPP1 was stored in aliquots of 1 μM in 1× TBS with 0.1% NP-40 at –80°C.

#### Purification of full-length ENPP3

Frozen cell pellets were resuspended in 10 mM HEPES pH 7.5, 150 mM sucrose and crushed in a bead mill (FisherScientific), 40 s on, 20 s off, for a total of 5 cycles. Following homogenization, the mixture was supplemented with Tris pH 7.5 to 50 mM, NaCl to 150 mM, imidazole to 10 mM, and cOmplete protease inhibitor cocktail (Roche). The mixture was centrifuged at 4 °C at 20,000g for 1 h and the supernatant was filtered through a .45 μm syringe filter. The filtered supernatant was then purified and stored according to the protocol described for secreted ENPP3.

#### Mouse models

C57BL/6J (Stock #000664) and C57BL/6J-Sting1gt/J (Stock #017537) mice were purchased from the Jackson Laboratory. *Enpp3*^−/−^ and *Enpp3*^H329A^ mice were generated and characterized in house and bred with C57BL/6J-Sting1gt/J to generate *Enpp3*^H329A^ × *Sting*^−/−^ and *Enpp3*^−/−^ × *Sting*^−/−^ mice. Male and female mice were included in every experiment, unless otherwise noted. Mice were maintained at Stanford University in compliance with the Stanford University Institutional Animal Care and Use Committee regulations. All procedures were approved by the Stanford University Administrative Panel on Laboratory Animal Care.

Generation of mice: First, single-guide RNAs (sgRNAs) were designed against the H329A locus in exon 11 of mouse Enpp3 using publicly available design tools (13). The sgRNA was then complexed with Alt-R S.p. Cas9 nuclease (Integrated DNA Technologies) as a ribonucleoprotein (RNP) particle. We then designed a donor sequence based on mouse Enpp3 to serve as the template for homologous recombination (Figure S3). The 200 nucleotide-long donor sequence contained blocking mutations near the PAM sequence to prevent repeated editing.

The donor sequence was then synthesized as single-stranded DNA (Integrated DNA Technologies). The RNP particles and donor template were microinjected into the pronuclei of one-cell embryos from C57BL/6 WT or ENPP1^H362A^ mice, which were then implanted into pseudo-pregnant mice. The injections were carried out by Stanford Transgenic, Knockout and Tumor Model Center. The initial litters of mice were backcrossed onto the WT C57BL/6 background and sequenced. Mice bearing the H329A mutation were crossed to each other to establish the ENPP3 H32A strain. Mice bearing a +1 frameshift mutation were crossed to each other to establish the ENPP3 KO strain.

#### Oligonucleotides for generating ENPP3^−/−^ and ENPP3^H329A^ mice

**Table:** 

Name	Sequence (5′>3′)

ENPP3 Exon 11 sgRNA	tggaagagcctgattctgca
ENPP3 Exon 11 Sequencing Fwd	tgaactggggcaggaatgaca
ENPP3 Exon 11 Sequencing Rev	cccaggacacagcacagaaa
ENPP3 H329A donor sequence for homologous recombination	gctcatgatcccaaattattaatgtctccctttgtttcaattttacctgtagacccagtttttatacca tctatgtggaagagcctgattctgcaggagcttcgagtggaccagtcagtgctggagtaagatgg ggttttcttgctggttttgtttgtttgtttgtttctcaacagatggaaaatcatttaggcctaacc

#### Orthotopic E0771 tumor model

Five-to nine-week-old female mice were used for all experiments, and all tumor inoculations were performed with PBS as the vehicle. Mice of the indicated genotype were inoculated with 250,000 E0771 cells suspended in 100 μL into the fifth mammary fat pad. Tumor volumes were recorded and analyzed with a generalized estimation equation (width*height*height/2). Mice were euthanized when tumor volumes reached 1000 mm^3^. Pairwise comparisons of the treatment groups at each time point were done by using post hoc tests with a Tukey adjustment for multiple comparisons. Animal death was plotted in a Kaplan–Meier curve with GraphPad Prism 9.3.1, and statistical significance was assessed by log rank Mantel–Cox test.

#### E0771 metastasis model

50,000 E0771.LMB.PuroR cells were per mouse injected into the indicated genotype via the tail vein using a 26-gauge needle. On day 30, lungs were harvested from mice, homogenized and plated in the presence of 1 μg/μL puromycin for 9 days to select for metastatic E0771 cells. Metastasis of each mouse was scored following staining of the E0771 cells by methylene blue and image analysis via Fuji ImageJ of blue-stained areas.

#### B16 metastasis model

200,000 B16 cells were per mouse injected into the indicated genotype via the tail vein using a 26-gauge needle. On day 20, lungs were harvested from mice and fixed in formaldehyde. Surface metastases were counted and graphed using an unpaired t test to calculate significance.

### QUANTIFICATION AND STATISTICAL ANALYSIS

All statistical calculations are annotated in the figure captions and were performed with the use of GraphPad Prism software. Graphs show means and standard deviation (±SD) or individual data points as indicated in the figure legends. Group size and experimental details are described in the figure legends. Statistical significance is calculated as 0.1234 (ns), 0.0332 (*), 0.0021 (**), 0.0002 (***).

## Supplementary Material

Supplemental Information

Supplemental Table 1

Supplemental information can be found online at https://doi.org/10.1016/j.celrep.2024.114209.

## Figures and Tables

**Table:** KEY RESOURCES TABLE

REAGENT or RESOURCE	SOURCE	IDENTIFIER

Chemicals, peptides, and recombinant proteins		

DMEM	Corning	Cat#MT10013CV
RPMI 1640	Corning	Cat#10–040-CV
FBS	Atlanta Biologics	Cat#S11150
Penicillin/streptomycin	ThermoFisher	Cat#15140163
HEPES (1 M)	Gibco	Cat#15–630-080
^32^P-labelled 2^’^3^’^-cGAMP	This paper	N/A
2^’^3^’^-cGAMP	Ritchie et al.^41^	N/A
Recombinant sscGAS	Ritchie et al.^41^	N/A
[α−^32^P]ATP, 250 μCi	Revvity	BLU003H250UC
GTP, disodium salt hydrate	Sigma Millipore	Cat#G8877–1G
Herring testes DNA	Sigma Millipore	Cat#D6898–1G
HP-TLC silica gel aluminum	EMD Millipore	Cat#1.05548.0001
Polyphosphate:AMP phosphotransferase (PAP)	Mardjuki et al.^10^	N/A
Myokinase	Sigma Millipore	Cat#M3003–2.5KU
His-Pur cobalt resin	ThermoFisher	89964
iScript cDNA synthesis kit	BioRad	1708890
Phusion High-Fidelity DNA polymerase	NEB	M0530S
Recombinant mouse ENPP2	R&D Systems	6187-EN-010
Recombinant human ENPP3	AcroBiosystems	EN3-H52H4–100ug
Recombinant mouse ENPP4	R&D Systems	8996-EN-020
Recombinant human ENPP5	R&D Systems	8655-EN-020
M-PER Mammalian Protein Extraction Reagent	Thermo Scientific	78501

Critical commercial assays		

CellTiterGlo	Promega	Cat#G8461
2^’^3^’^ cGAMP ELISA	Cayman Chemical	501700
UMP/CMP-Glo^™^ Glycosyltransferase Assay	Promega	VA1130

Experimental models: Cell lines		

293T cGAS *ENPP1*^−/−^ cells	Carozza et al.^8^	N/A
EO771	ATCC	CRL-3461; RRID:CVCL_GR23
EO771.lmb.PuroR	Wang et al.^12^	CRL-3405; RRID:CVCL_B0A2
B16-F10	ATCC	CRL-6475; RRID:CVCL_0159

Experimental models: Organisms/strains		

WT C57BL/6J mice	Jackson Laboratory	RRID:IMSR_JAX:000664
*STING–*^/–^ mice (C57BL/6J-Sting1gt/J)	Jackson Laboratory	RRID:IMSR_JAX:025805
C57BL/6J *Enpp3*^−/−^	This paper	N/A
C57BL/6J *Enpp3*^H329A^	This paper	N/A
C57BL/6J *Enpp3*^H362A^	Carozza et al.^8^	N/A
C57BL/6J *Enpp3*^H329A^ × *Sting*^−/−^	This paper	N/A
C57BL/6J *Enpp1*^H362A^ × *Enpp3*^−/−^	This paper	N/A

Oligonucleotides		

Full length mENPP3 forward	This paper	CTACGGGAACAATGGATTCCAG
Full length mENPP3 reverse	This paper	CATTCAAATAATGGTTTCAAACGTGGGCAGATACGTC
mENPP3 H329A mutation forward	This paper	CCTGATTCTGCAGGGGCGTCGAGTGGACCAGTC
mENPP3 H329A mutation reverse	This paper	GACTGGTCCACTCGACGCCCCTGCAGAATCAGG
mENPP3 Exon 11 sgRNA	This paper	Tggaagagcctgattctgca
mENPP3 Exon 11 sequencing forward	This paper	tgaactggggcaggaatgaca
mENPP3 Exon 11 Sequencing reverse	This paper	cccaggacacagcacagaaa
mENPP3 H329A donor sequence for homologous recombination	This paper	gctcatgatcccaaattattaatgtctccctttgtttcaattttacctgtagaccc agtttttataccatctatgtggaagagcctgattctgcaggagcttcgagtgg accagtcagtgctggagtaagatggggttttcttgctggttttgtttgtttgtttgt ttctcaacagatggaaaatcatttaggcctaacc

Software and algorithms		

GraphPad Prism	GraphPad	https://www.graphpad.com
ImageJ	NIH	https://imagej.nih.gov/ij/download.html
Pymol	Schrödinger, Inc.	https://www.pymol.org/2/

Other		

Superose 6 Increase 10/300 GL column	GE Healthcare	GE17–5173-01
HiTrap Q HP anion exchange	Cytiva	17115301
Amersham Typhoon 5 Imager	Cytiva	29187191
Storage Phosphor Screen	Cytiva	28956476
Bead Mill 24 Homogenizer	FisherScientific	15–340-163

## Highlights

- ENPP3 is the only other major metazoan cGAMP hydrolase under homeostatic conditions
- ENPP3 has a cancer and tissue expression pattern distinct from that of ENPP1
- ENPP3 is an innate immune checkpoint that should be targeted for the treatment of cancer
