# Supplementary material for: Identification of the extracellular membrane protein ENPP3 as a major cGAMP hydrolase and innate immune checkpoint
Source: Cell Rep. Author manuscript; Available in PMC 2026 Jun 15. (PMC13268100; doi:10.1016/j.celrep.2024.114209)
Supplement: Supplemental Information [file NIHMS2147837-supplement-Supplemental_Information.pdf]

Cell Reports, Volume *43*

Supplemental information

Identification of the extracellular membrane protein ENPP3 as a major cGAMP hydrolase and innate immune checkpoint

Rachel Mardjuki, Songnan Wang, Jacqueline Carozza, Bahar Zirak, Vishvak Subramanyam, Gita Abhiraman, Xuchao Lyu, Hani Goodarzi, and Lingyin Li

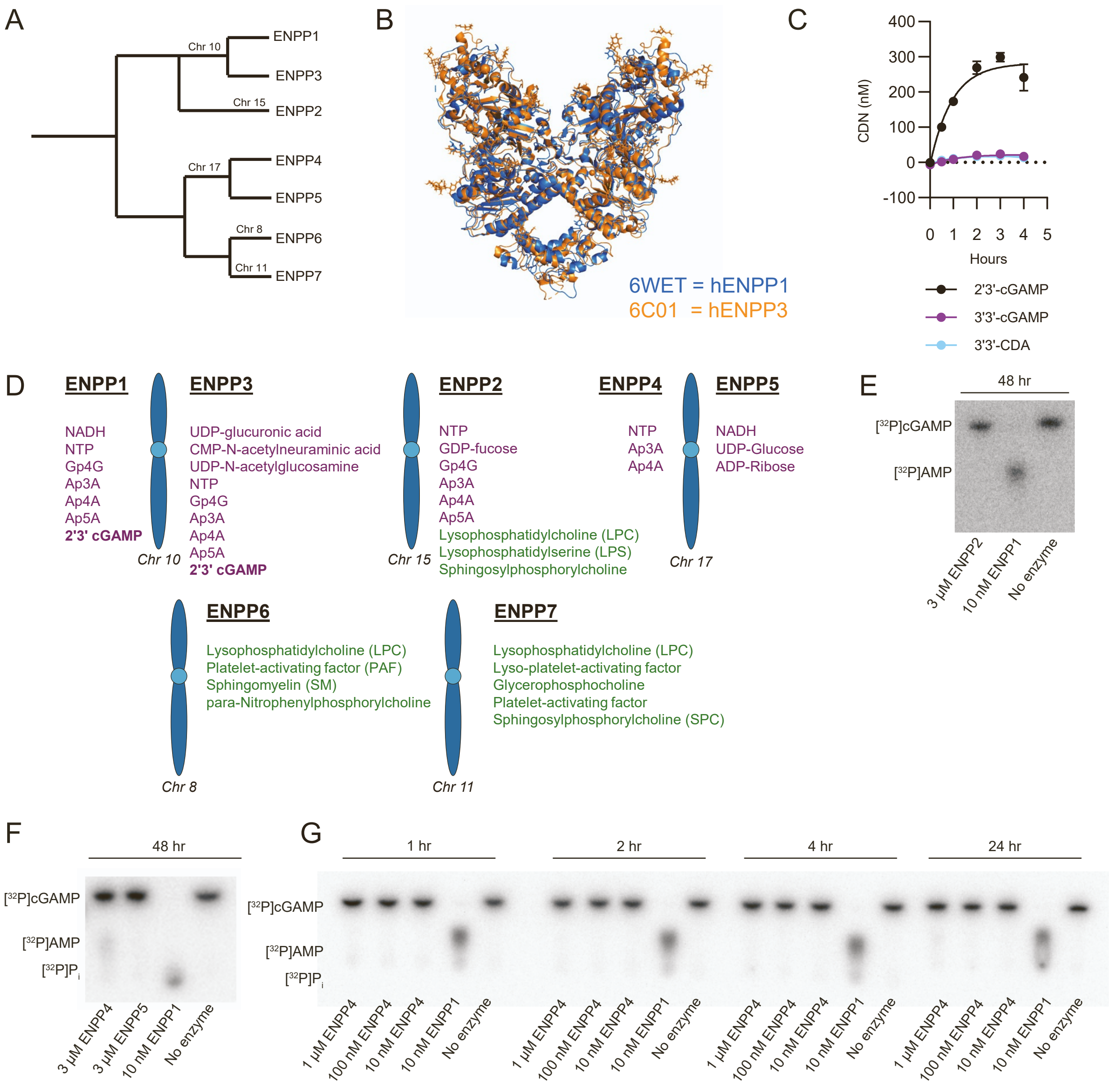

Supplemental Figure 1: Homology among the ENPP family, related to Figure 1.
(A) Evolutionary tree of the mouse ENPP family based on genetic similarity generated by Seaview.
(B) Overlaid crystal structures of human ENPP1 (PDB 6WET) and human ENPP3 (PDB 6C01).
(C) Kinetics of degradation of indicated cyclic dinucleotide by recombinant ENPP3.
(D) Schematic depicting the substrates each member of the ENPP family is known to cleave. Purple text indicates a nucleotide-base substrate; green text indicates a lipid-base substrate.
(E-G) Degradation of ^{32}P-labeled cGAMP by recombinant ENPP1, ENPP2, ENPP4 and ENPP5.

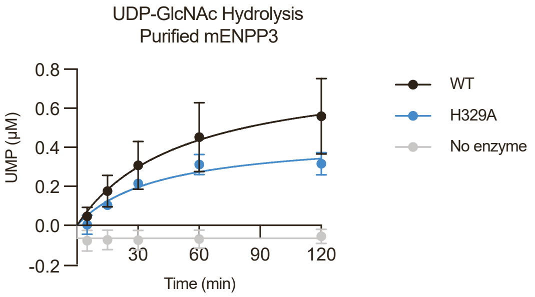

Supplemental Figure 2: **Kinetics of hydrolysis of UDP-GlcNAc by purified mouse WT and H329A ENPP3, related to Figure 3**. Mean ± SD (n = 4 for WT, n = 2 for H329A).

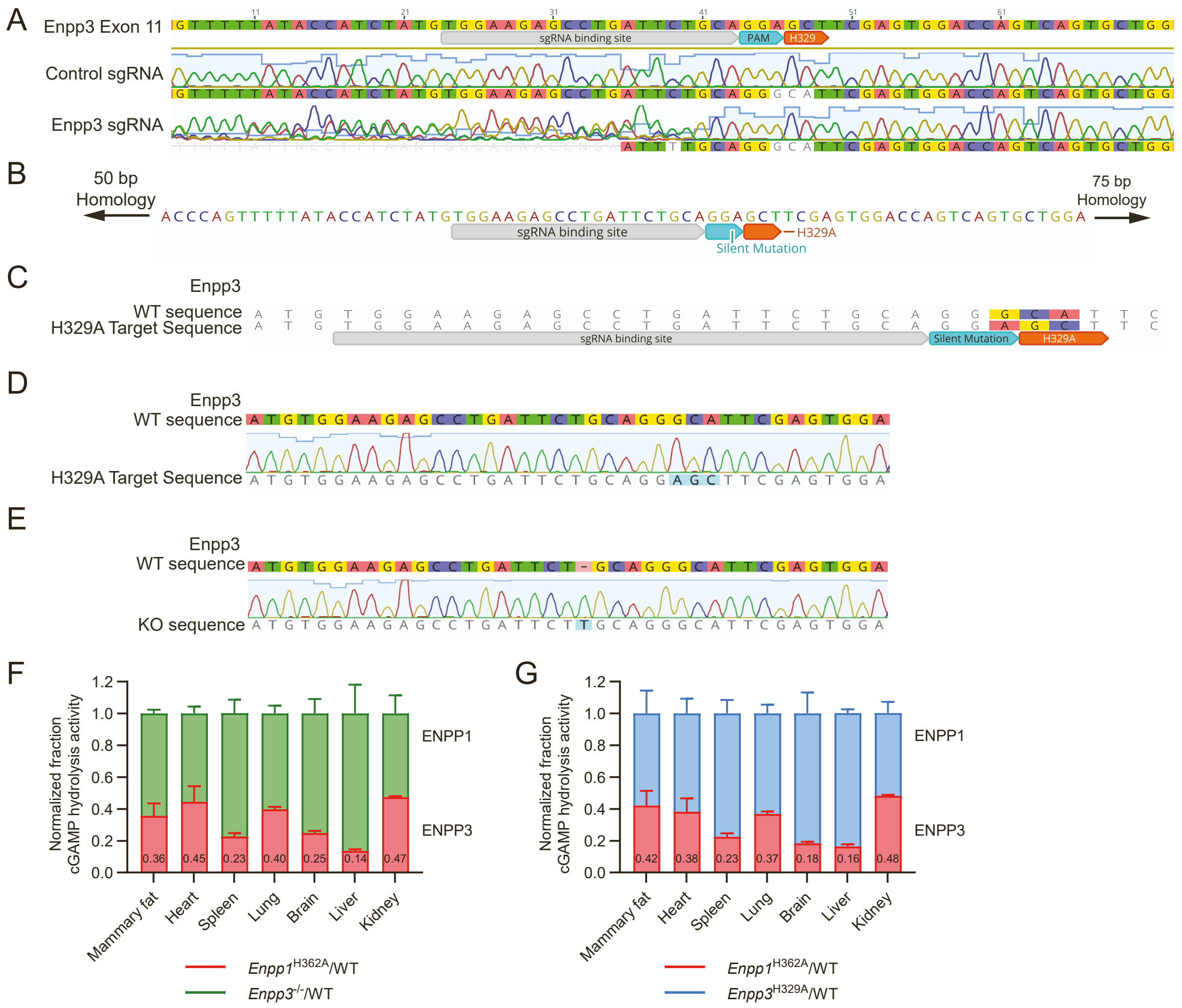

Supplemental Figure 3: **Generation of *Enpp3*^{H329A} and *Enpp3*$^{-/-}$ mice, related to Figure 3.**
(A) A single guide RNA (sgRNA) was designed to target the region near H329A in exon 11 of *Enpp3*. The sgRNA and Cas9 were introduced into 4T1 cells through lentiviral transduction. The cells were then sequenced to determine editing efficiency at the intended cleavage site.
(B) A homology arm was designed to generate the H329A point mutation through homologous recombination. A silent mutation was introduced downstream of H329 to prevent Cas9 from recognizing the PAM sequence following successful recombination.
(C) Schematic illustrating the three base pair changes incorporated following successful recombination of the homology arm.
(D) Schematic illustrating typical sequencing of genomic DNA from a homozygous *Enpp3*^{H329A} mouse.
(E) Schematic illustrating typical sequencing of genomic DNA from a homozygous *Enpp3*$^{-/-}$ mouse.
(F-G) Normalized activity of ENPP1 (from fraction of ENPP1^{H362A} activity as compared to WT in each indicated organ) and ENPP3 (from fraction of ENPP3^{H329A} or ENPP3$^{-/-}$ activity as compared to WT in each indicated organ) displayed in stacked bar graph. Mean ± SD; *n*=3 for all but ENPP3 genotypes where *n*=2.

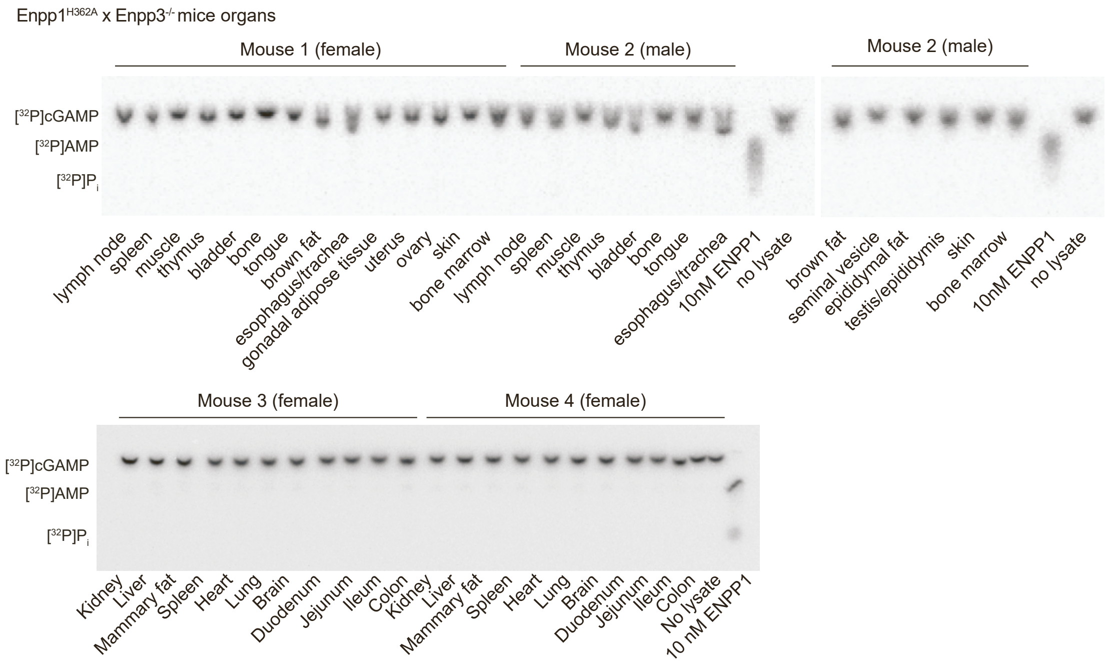

Supplemental Figure 4: cGAMP hydrolase activity in indicated organ lysate of *Enpp1*^{H362A} x *Enpp3*$^{-/-}$ mice, related to Figure 3.
Each reaction was supplemented with physiological divalent ions (pH 7.5, 37°C, 48 hours).

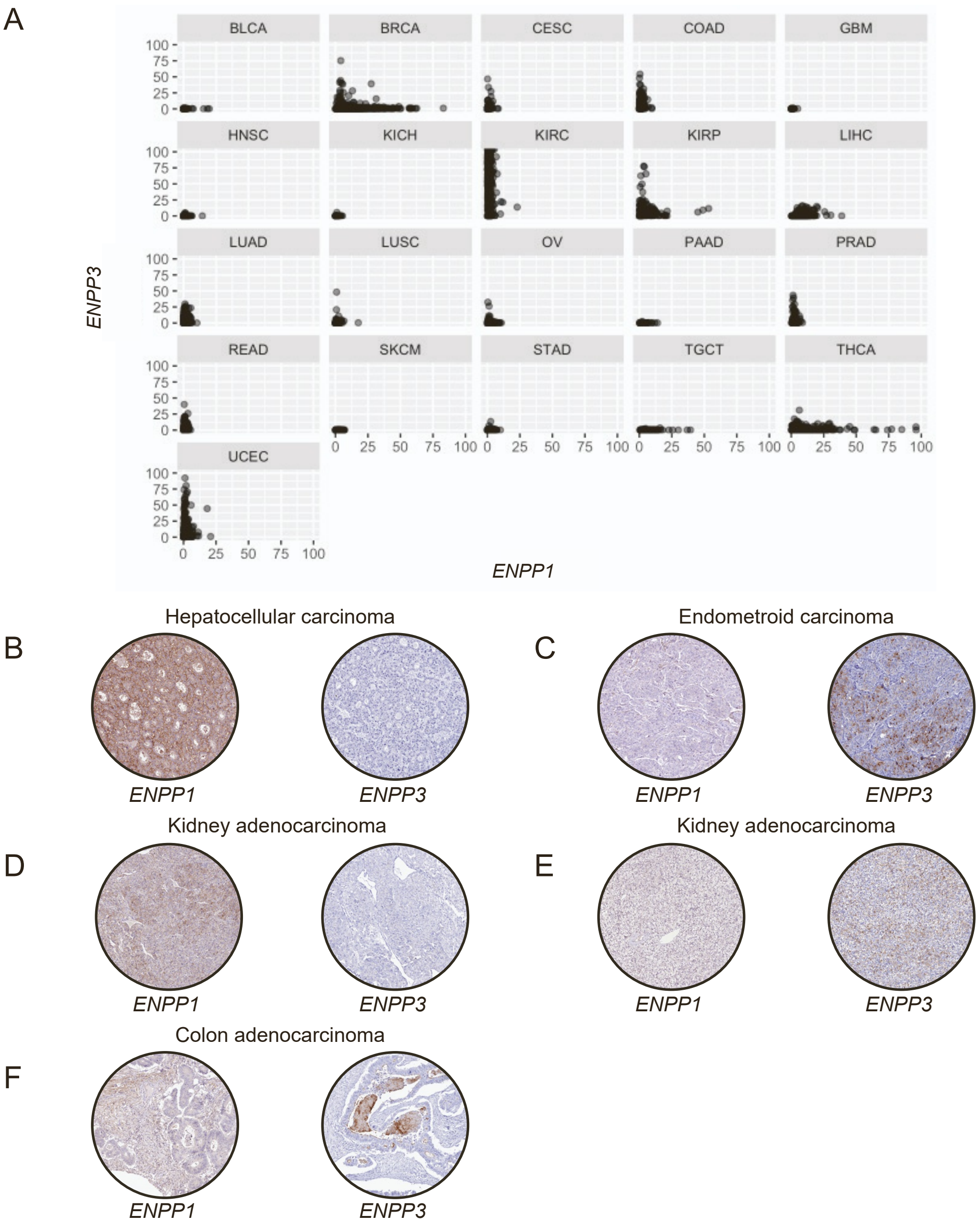

Supplemental Figure 5: ENPP1 vs. ENPP3 expression in human tumors, related to Figure 4.
(A) Quantification of expression of ENPP1 and ENPP3 from human cancer tissues in nTPM. Data from TCGA.
(B-F) Immunohistochemistry staining of ENPP1 and ENPP3 from human cancer tissues; sections were stained with hematoxylin (blue) and brown staining indicates where an antibody labelled with DAB (3,3'-diaminobenzidine) bound either ENPP1 or ENPP3. Data from the Human Protein Atlas.
